# Predicting the Lattice Thermal Conductivity in Nitride Perovskite LaWN_3_ from ab initio Lattice Dynamics

**DOI:** 10.1002/advs.202205934

**Published:** 2023-01-22

**Authors:** Zhen Tong, Yatian Zhang, Alessandro Pecchia, ChiYung Yam, Liujiang Zhou, Traian Dumitrică, Thomas Frauenheim

**Affiliations:** ^1^ Shenzhen JL Computational Science and Applied Research Institute Shenzhen 518131 China; ^2^ Beijing Computational Science Research Center Beijing 100193 China; ^3^ Bremen Center for Computational Materials Science University of Bremen 28359 Bremen Germany; ^4^ CNR‐ISMN Via Salaria Km 29.300, Monterotondo Rome 00017 Italy; ^5^ School of Physics University of Electronic Science and Technology of China Chengdu 610054 China; ^6^ Department of Mechanical Engineering University of Minnesota Minnesota 55455 USA

**Keywords:** *ab initio* calculations, lattice thermal conductivity, nitride perovskites, temperature renormalization, glassy systems

## Abstract

Using a density functional theory‐based thermal transport model, which includes the effects of temperature (*T*)‐dependent potential energy surface, lattice thermal expansion, force constant renormalization, and higher‐order quartic phonon scattering processes, it is found that the recently synthesized nitride perovskite LaWN_3_ displays strong anharmonic lattice dynamics manifested into a low lattice thermal conductivity (*κ*_*L*_) and a non‐standard *κ*_*L*_∝*T* ^−0.491^ dependence. At high *T*, the departure from the standard *κ*_*L*_∝*T* ^−1^ law originates in the dual particle‐wave behavior of the heat carrying phonons, which includes vibrations tied to the N atoms. While the room temperature *κ*_*L*_=2.98 W mK^‐1^ arises mainly from the conventional particle‐like propagation of phonons, there is also a significant atypical wave‐like phonon tunneling effect, leading to a 20% glass‐like heat transport contribution. The phonon broadening effect lowers the particle‐like contribution but increases the glass‐like one. Upon *T* increase, the glass‐like contribution increases and dominates above *T* = 850 K. Overall, the low *κ*_*L*_ with a weak *T*‐dependence points to a new utility for LaWN_3_ in energy technology applications, and motivates synthesis and exploration of nitride perovskites.

## Graphical abstract

Although LaWN_3_ is a crystal, it displays an unusual glass‐like transport channel, which contributes 20% to lattice thermal conductivity at room temperature. Above 850 K, glass‐like transport through wave‐like phonon tunneling dominates over the standard particle‐like transport. The ab initio calculations of low lattice thermal conductivity predict a new utility of LaWN_3_ for the energy technology.

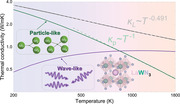

## 1 Introduction

The recently synthesized Lanthanum Tungsten Nitride (LaWN_3_),^[^ ^1^ ^]^ a new crystal with rhombohedral (*R*3*c*, Space Group 161) symmetry first predicted by *ab* *initio* calculations,^[^ ^2^ ^]^ extends to nitrides the family of perovskite structured materials with ABX_3_ stoichiometry, beyond oxides, halides, and chalcogenides. So far, LaWN_3_ attracted great attention due to its strong piezoelectricity^[^ ^1^ ^]^ and its good prospects for integration in a variety of nitride‐based semiconductor platforms including aluminum nitride^[^ ^3^ ^]^ and gallium nitride^[^ ^4^ ^]^ used in wireless communication networks.

Perovskites are of vast technological importance for the field of energy.^[^ ^5^ ^]^ They are typically known for exhibiting low lattice thermal conductivity (*κ*_*L*_). Ultra‐low *κ*_*L*_ values have been occasionally reported^[^ ^6^, ^7^, ^8^, ^9^, ^10^, ^11^, ^12^, ^13^ ^]^ and associated to diverse microscopic conductivity‐lowering mechanism. For example, Lee et al.^[^ ^6^ ^]^ identified cluster‐rattling in inorganic halide perovskite, Osei‐Agyemang et al.^[^ ^13^ ^]^ found strong anharmonicity in CaZrSe_3_, while Sun et al.^[^ ^9^ ^]^ found atomic tunneling (dynamic disorder) through elastic and inelastic scattering measurements in chalcogenide perovskite BaTiS_3_. A comprehensive theoretically investigation^[^ ^12^ ^]^ found *κ*_*L*_ in halides to be typically one order of magnitude lower than in oxides. Unfortunately, to date there is no thermal conductivity data for the new nitride perovskite LaWN_3_.

Building a predictive theoretical model for *κ*_*L*_ is technically challenging as it requires an *ab* *initio* level of accuracy for the description of the interatomic interactions not only at ground state (0 K) but also at finite temperature (*T*). This is because the *T*‐dependency of phonon frequency and anharmonic force constants proved important for the accurate determination of *κ*_*L*_ in strongly anharmonic materials.^[^ ^10^, ^12^, ^14^, ^15^, ^16^, ^17^ ^]^ Moreover, the traditional particle‐like treatment of phonons may not be sufficient for describing thermal transport in the complex LaWN_3_ structure. Indeed, the wave‐particle duality of phonons is not peculiar to nanostructures but holds in bulk crystals as well.^[^ ^18^ ^]^ In this respect, recent studies found that the thermal properties of ternary crystals with strong anharmonicity and dense band structure, including Tl_3_VSe_4_,^[^ ^16^ ^]^ CsPbBr_3_,^[^ ^19^ ^]^ and La_2_Zr_2_O_7_,^[^ ^20^ ^]^ display the fingerprints of the wave‐like behavior of phonons. Specifically, the wave‐like tunneling between closely spaced phonon branches can lead to a glass‐like heat transport component.

In this work, we unveil the lattice thermal transport of LaWN_3_ by means of density functional theory (DFT) calculations and a generalized thermal transport formulation,^[^ ^19^, ^21^ ^]^ which captures both the conventional particle‐like behavior and wave‐like behavior of phonons. The effect of *T* is rigorously accounted for by considering the *T*‐dependent potential energy surface, the lattice thermal expansion, the force constant renormalization extracted from the trajectory of *ab* *initio* molecular dynamics (AIMD) simulations at finite *T*, and the inclusion of three‐phonon (3ph) and four‐phonon (4ph) scatterings (in‐house implementations). The wave‐like (or glass‐like) and particle‐like components are evaluated separately and the dominant phonon behavior is elucidated over a wide temperature range.

Before we begin, we would like to note that the basic anharmonic lattice dynamics computational framework,^[^ ^22^, ^23^ ^]^ implemented extensively in ShengBTE,^[^ ^24^ ^]^ Phono3py,^[^ ^25^ ^]^ D3Q,^[^ ^26^ ^]^ and almaBTE,^[^ ^27^ ^]^ predicts the phonon thermal conductivity with the ground 0 K harmonic description of the phonon frequency and phonon lifetime by capturing only 3ph scatterings.^[^ ^28^, ^29^, ^30^, ^31^ ^]^ Inclusion of the 4ph scattering in this ground picture, which has been implemented as an extension to ShengBTE,^[^ ^32^ ^]^ has been proved to be insufficient for predicting thermal conductivity of compounds with strong high‐order anharmonicity.^[^ ^16^, ^19^, ^20^, ^21^, ^33^ ^]^ Building on this 0 K ground interatomic force constants (G‐IFCs) approach, further progress was brought by using the self‐consistent phonon approximation (SCPH),^[^ ^34^ ^]^ which considers the phonon frequency renormalization by capturing the effect of higher‐order anharmonic phonon‐phonon interactions, and the inclusion of *T*‐dependent effective potential (TDEP),^[^ ^35^ ^]^ which involves the *T*‐dependent potential energy surface, lattice thermal expansion, and force constant renormalization. These methods started to be integrated with ab initio calculations to gain an accurate estimation of phonon frequency and phonon thermal conductivity. The standard SCPH^[^ ^34^ ^]^ and TDEP^[^ ^35^ ^]^ packages only consider the 3ph scatterings when computing the phonon lifetime. The wave‐like contribution is implemented in SCPH with considering 3ph scattering, but not in TDEP. As in Refs.[16, 36, 37], here we are relying on our in‐house implementation for including 4ph scattering in the phonon lifetime and the wave‐like contributions based on the theory level of renormalized phonons and anharmonic IFCs.

**Figure 1:**
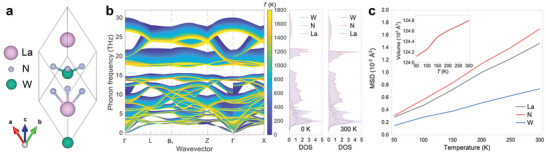
a) Crystal structure of LaWN_3_ with *R*3*c* symmetry. Lanthanum, tungsten, and nitrogen atoms are represented as red, green, and gray spheres, respectively. b) Calculated temperature‐dependent phonon dispersions of LaWN_3_ from 0 to 1800 K and the atom decomposed phonon density of states at 0 and 300 K, respectively. The shaded red areas depict the total density of states. The phonon dispersion at 0 K is calculated from DFT using Phonopy.^[^ ^38^ ^]^ The finite temperature dispersions are calculated from AIMD with TDEP.^[^ ^35^ ^]^ c) Calculated temperature dependent atomic mean square displacements (MSD) of LaWN_3_ along the [111] direction for La (gray), N (red), and W (blue) atoms. The inset is the temperature dependent volume of LaWN_3_.

**Figure** **1**a displays the computed 10‐atom unit cell of LaWN_3_ while Figure 1b shows the *T*‐dependent phonon dispersion from 0 to 1800 K, in which the effect of lattice thermal expansion shown in the inset of Figure 1c, was taken into account. The phonon dispersion shows no signs of lattice instabilities but densely packed phonon branches with significant phonon broadening. The high‐lying modes, above ≈7.5 THz, are dominated by the vibrations of N atoms, as it is revealed by the atom‐decomposed phonon density of states in Figure 1b right. Further, it can be seen in Figure 1c that the low‐mass N atoms undergo the largest mean square displacements (MSDs) due to its smallest mass and vertex sharing of the octahedron in LaWN_3_ around their equilibrium position, leading to the significant phonon softening, Figure 1b. The low‐lying acoustic modes, bellow ≈7.5 THz, are attributed mainly to La and W. While both atoms have somewhat smaller MSDs, Figure 1c, according to AIMD their vibration still give significant phonon broadening, Figure 1b.

By working in the framework of the Wigner formalism of quantum mechanics,^[^ ^39^ ^]^ Simoncelli et al.^[^ ^19^, ^40^ ^]^ derived a lattice thermal conductivity $\kappa_{L}^{\alpha \beta }$ expression comprising separate particle‐like ($\kappa_{p}^{\alpha \beta }$) and glass‐like (coherence, $\kappa_{c}^{\alpha \beta }$) terms

(1) $$\kappa_{L}^{\alpha \beta }=\kappa_{p}^{\alpha \beta }+\kappa_{c}^{\alpha \beta }$$

where *α* and *β* are indexing the Cartesian directions. Consider the phonon mode indexed by wave vector **q** and branch *s*. The particle‐like contribution coinciding with the standard Peierls–Boltzmann thermal conductivity results from the diagonal (*s* = *s*′) terms of the Wigner heat‐flux operator,^[^ ^19^ ^]^ and it is expressed as

(2) $$\kappa_{p}^{\alpha \beta }=\frac{1}{VN_{q}}\sum_{qs}C_{q}^{s}v_{q,\alpha }^{s}v_{q,\beta }^{s}\tau_{q}^{s}$$

The off‐diagonal (*s* ≠ *s*′) terms give rise to

(3) $$\begin{array}{cc} \kappa_{c}^{\alpha \beta }= & \frac{\hbar^{2}}{k_{B}T^{2}VN_{q}}\sum_{q}\sum_{s\neq s^{\prime }}\frac{\omega_{q}^{s}+\omega_{q}^{s^{\prime }}}{2}v_{q,\alpha }^{s,s^{\prime }}v_{q,\beta }^{s,s^{\prime }}\\ & \times \frac{\omega_{q}^{s}n_{q}^{s}\left(n_{q}^{s}+1\right)+\omega_{q}^{s^{\prime }}n_{q}^{s^{\prime }}\left(n_{q}^{s^{\prime }}+1\right)}{4{\left(\omega_{q}^{s}-\omega_{q}^{s^{\prime }}\right)}^{2}+{\left(\Gamma_{q}^{s}+\Gamma_{q}^{s^{\prime }}\right)}^{2}}\times \left(\Gamma_{q}^{s}+\Gamma_{q}^{s^{\prime }}\right) \end{array}$$

the coherences' term^[^ ^19^ ^]^ that describes the tunneling between phonon branches *s* and *s*′. In these expressions, ℏ, *k*_*B*_, *V*, and *N*_**q**_ are the reduced Plank constant, the Boltzmann constant, the volume of the unit cell, and the number of sampled phonon wave vectors in the first Brillouin zone, respectively. $C_{q}^{s}$, $v_{q}^{s}$, $\tau_{q}^{s}$, $\omega_{q}^{s}$, $v_{q}^{s,s^{\prime }}$, $\Gamma_{q}^{s}$ ($\tau_{q}^{s}$=1/$\Gamma_{q}^{s}$) are the heat capacity, the group velocity, the lifetime, the phonon frequency, the velocity operator, and the scattering rate of a phonon mode, respectively. $n_{q}^{s}={\left[\exp\left(\hbar \omega_{q}^{s}/\left(\kappa_{B}T\right)\right)-1\right]}^{-1}$ is the equilibrium Bose–Einstein distribution. More details of the derivations of the terms are provided in the Supporting Information. For accuracy, both 3ph ($\Gamma_{q}^{s,3\mathrm{ph}}$) and 4ph ($\Gamma_{q}^{s,4\mathrm{ph}}$), namely $\Gamma_{q}^{s}=\Gamma_{q}^{s,3\mathrm{ph}}+\Gamma_{q}^{s,4\mathrm{ph}}$, are included when computing *κ*_p_ and *κ*_c_.

## 2 Results and Discussion

**Figure 2:**
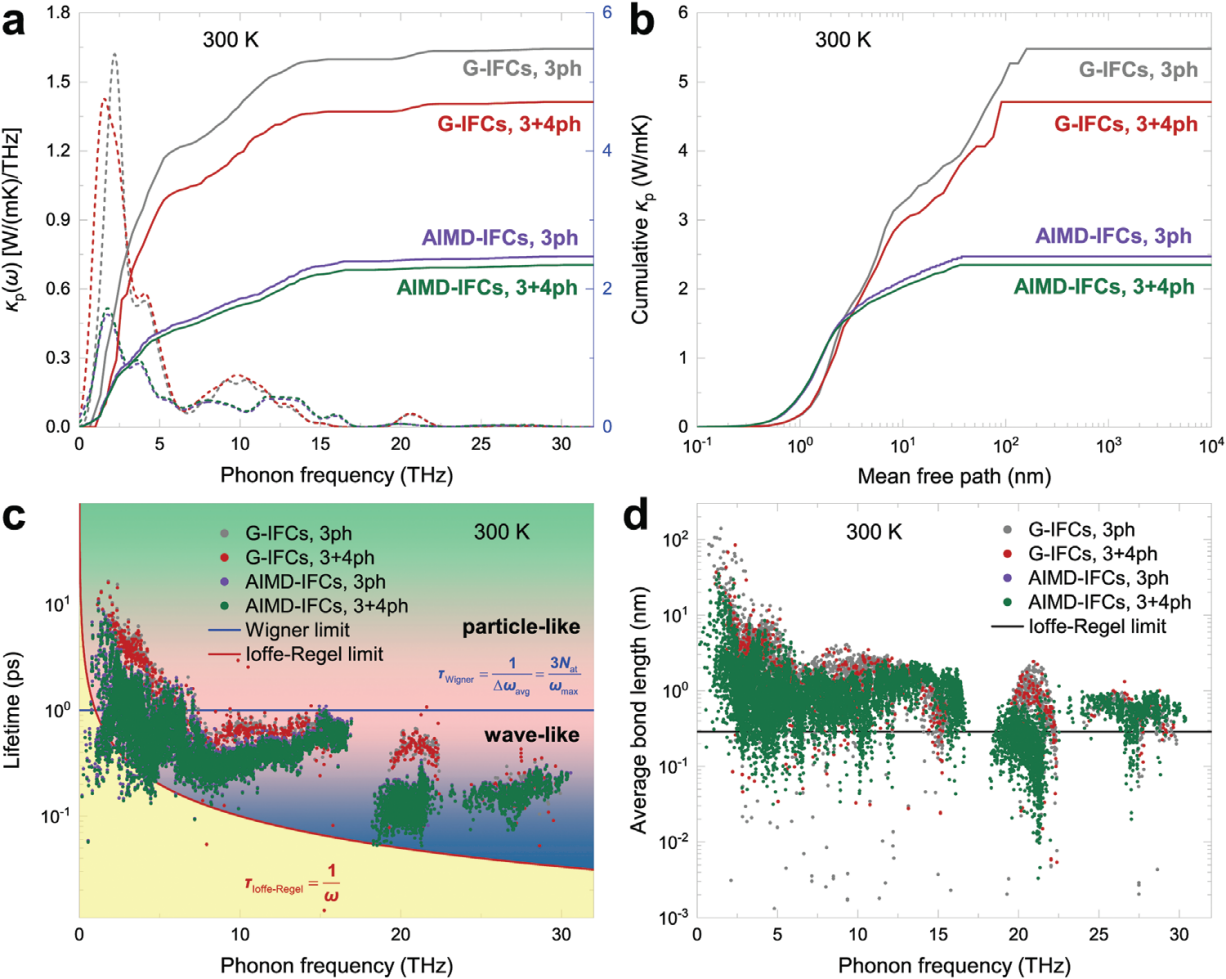
Phonon thermal (particle‐like contribution) properties of LaWN_3_ at 300 K. a) Comparison of cumulative (solids lines) and differential (dashed lines) *κ*_p_ calculated using the interatomic force constants obtained at ground 0 K (G‐IFCs) and finite *T* (AIMD‐IFCs). The different theory levels are shown in different line color: gray for G‐IFCs with 3ph scattering, red for G‐IFCs with 3+4ph scattering, violet for AIMD‐IFCs with 3ph scattering, and green for AIMD‐IFCs with 3+4ph scattering. b) Same as (a) but for the mean free path cumulative *κ*_p_. c) Comparison of phonon lifetime. The solid‐red line is the Ioffe–Regel limit^[^ ^41^ ^]^ in time, *τ*_Ioffe‐Regel_ = 1/*ω*. The solid‐blue line is the Wigner limit,^[^ ^40^ ^]^ corresponding to a phonon lifetime equal to the inverse of the average interband spacing Δ*ω*_ave_, namely *τ*_Wigner_ = 1/Δ*ω*_ave_ = 3*N*_at_/*ω*_max_ where *ω*_max_ is the maximum phonon frequency and 3*N*_at_ is the number of phonon bands. d) Same as (c) but for mean free path.

**Figure 3:**
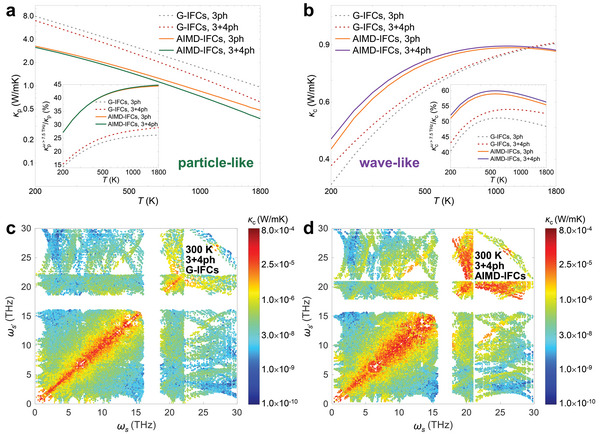
a) *κ*_p_ and b) *κ*_c_ vs *T* calculated using the G‐IFCs and AIMD‐IFCs based on 3ph and 3+4ph scattering. The insets show the thermal conductivity contribution for phonons with frequency >7.5 THz. The *κ*_c_ terms associated with various pairs of phonon frequencies (*ω*_*s*_ and $\omega_{s^{\prime }}$) at 300 K from c) G‐IFCs and d) AIMD‐IFCs with 3+4ph scattering.

With the above ab initio lattice model and the unified *κ*_*L*_ formula for particle‐like and wave‐like transport, we now explore the mechanism of lattice conduction. First, we note that LaWN_3_ displays significant anharmonicity, which impacts *κ*_p_. Using G‐IFCs, it can be seen that by considering both the 3ph and 4ph scattering, the converged *κ*_p_ is lowered by 0.8 W mK^‐1^ from the 5.48 W mK^‐1^ value computed with only 3ph scattering at 300 K. Further using the AIMD‐IFCs with 3ph scattering at 300 K leads to a ≈ 50 % reduction, bringing *κ*_p_ down to 2.47 W mK^‐1^ for 3ph scattering and even to a lower value of 2.34 W mK^‐1^ when involving 3+4ph scattering. We can see that the AIMD‐IFCs with 3ph scatterings have strong effect on the thermal conductivity compared to the G‐IFCs in the case of both 3ph and 3+4ph scattering, while it decreases little after including the 4ph scattering (3+4ph), which indicates the importance of considering the temperature normalization on the IFCs when predicting the thermal conductivity of LaWN_3_. It should be noted that the plots of *κ* in this work are the average value of the three different crystallographic directions since LaWN_3_ displays a very weak anisotropy of the *κ* (see Figure S1, Supporting Information). Phonons of frequency below ≈15 THz (thus also the vibrational states of the N atoms) are mainly contributing to *κ*_p_. These can be seen in **Figures** **2**a,b, which plot the accumulative *κ*_p_ with respect to phonon frequency and phonon mean free path (MFP), respectively. The *T* renormalization brings reductions in the phonon lifetimes (*τ*) and MFPs, as shown in Figures 2c,d, respectively. These reductions can be ultimately traced back to the enhancement of the phonon scattering phase space and the reduction of the phonon group velocity with increasing *T* (see Figure S2, Supporting Information). At high *T*, there is a significant *κ*_p_∝*T* ^−1^ decrease, **Figure** **3**a, and the accounting for the phonon broadening remains important. The inset of Figure 3a demonstrates that the broadened vibrational modes tied to the N atoms (i.e., modes with frequencies >7.5 THz) are bringing significant contributions to *κ*_p_, especially in the very high *T* regime.

Second, we identify the signature of a wave‐like tunneling behavior in the heat carrying phonons. In Figure 2c, we marked with horizontal lines both the Wigner limit *τ*_Wigner_ (when the phonon lifetime equals the inverse of the average interband spacing, i.e., *τ*_Wigner_ = 1/Δ*ω*_ave_^[^ ^40^ ^]^) and the Ioffe‐Regel limit *τ*_Ioffe‐Regel_ (when the phonon lifetime equals the inverse of the phonon frequency, i.e., *τ*_Ioffe‐Regel_ = 1/*ω* ^[^ ^41^ ^]^). Phonons with *τ* > *τ*_Wigner_ (toward the green background) are those contributing to *κ*_p_. Unlike in a typical crystal, in LaWN_3_ there is a large population of phonons with *τ*_Ioffe‐Regel_ < *τ* < *τ*_Wigner_ (more phonon lifetimes at different *T* are provided in Figure S3, Supporting Information). These are the heat carrying wave‐like phonons which according to our calculations give *κ*_c_=0.64 W mK^‐1^ at *T*=300 K.

Interestingly, the *T*‐renormalization brings an enhancement to *κ*_c_, Figure 3b. For more insights into this feature, Figures 3c,d display the magnitude of *κ*_c_ component associated with the pairs of phonon branches (i.e., the term inside summation in Equation (3)), without and with *T*‐renormalization, respectively. The major contributors to *κ*_c_ are the dense phonon branches with very close frequencies. A comparison of the two figures indicates that throughout the considered frequency range, the *T*‐renormalization extends the coherences' coupling between modes with larger frequency differences. This enhancement is especially visible in high‐lying (above ≈7.5 THz) frequency region, which comprises the less dense vibrational branches assigned to the N group of atoms. The ratio of the *κ*_c_ with phonon frequency >7.5 THz to *κ*_c_, plotted *vs*. *T* in the inset of Figure 3b, indicates an over 50 % coherence's contribution to *κ*_c_ from the N group of atoms at all *T*.

**Figure 4:**
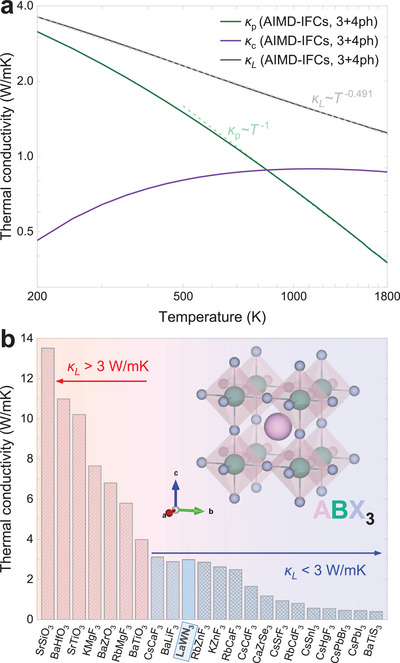
a) Thermal conductivity of LaWN_3_ *vs*. *T*: *κ*_p_ (green line), *κ*_c_ (purple line), and *κ*_*L*_ (gray line). The fitting of temperature dependence are presented with dash lines. b) Literature data for *κ*_*L*_ of other perovskite materials at 300 K. Oxide perovskite: SrSiO_3_, BaHfO_3_, SrTiO_3_, BaZrO_3_, and BaTiO_3_ from theoretical prediction.^[^ ^12^ ^]^ Halide perovskite: RbMgF_3_, CsCaF_3_, BaLiF_3_, RbZnF_3_, KZnF_3_, RbCaF_3_, CsCdF_3_, CsSrF_3_, RbCdF_3_, and CsHgF_3_ from theoretical prediction^[^ ^12^ ^]^ as well as CsSnI_3_, CsPbBr_3_, and CsPbI_3_ from experiment.^[^ ^6^ ^]^ Chalcogenide perovskite: BaTiS_3_ (experiment^[^ ^9^ ^]^) and CaZrSe_3_ (theoretical^[^ ^13^ ^]^).

**Figure** **4**a demonstrates the coexistence of *κ*_p_ and *κ*_c_ and that the dominant transport mechanism depends on *T*. While *κ*_p_ decreases strongly with *T*, *κ*_c_ exhibits the opposite trend. Above the crossover *T* of 850 K, the wave‐like thermal transport prevails and, at 1800 K becomes 3 times larger than *κ*_p_. This combination of behaviors leads to a nonstandard *κ*_*L*_∝*T* ^−0.491^ (gray dash line), which nevertheless is in harmony with the *κ*_*L*_ dependencies found in prior simulations of other complex and/or highly anharmonic crystals.^[^ ^16^, ^19^, ^20^, ^21^, ^33^ ^]^ For a broader view, in Figure 4b, we place our result in the context of other important perovskite materials. Our calculations assign LaWN_3_ to the select class of low thermal conductivity crystals,^[^ ^6^ ^]^ which are of enormous importance for advancing energy technologies such as thermoelectrics for energy harvesting,^[^ ^42^ ^]^ thermal barrier coatings for turbine blades,^[^ ^43^ ^]^ and heat‐assisted data storage devices.^[^ ^44^ ^]^

## 3 Conclusion

While LaWN_3_ is currently viewed as a revolutionary electronic material, our calculations predict its new utility for the energy technology. Using a complex and complete ab initio based computational framework, we find that crystalline LaWN_3_ exhibits strong anharmonicity which manifests into a low *κ*_*L*_ and a glass‐like thermal transport channel with significant contributions from the vibrations of N atoms. The recent synthesis of LaWN_3_^[^ ^1^ ^]^ makes possible to probe our theoretical predictions through carefully designed experiments.^[^ ^6^, ^9^ ^]^ Considering the important role of the coherences' coupling of vibrations involving N atoms found in LaWN_3_, it is likely that the glass‐like transport component will occur in other nitride perovskite crystals which currently are only theoretically predicted.^[^ ^2^ ^]^ In this respect, our predictions might motivate thermal property measurements in LaWN_3_ as well as synthesis of other nitride perovskites that might have a similarly exciting set of properties.

## 4 Experimental Section

### Ab Initio Calculations

Their ab initio calculations were carried out using projector‐augmented‐wave (PAW)^[^ ^45^ ^]^ method with the Perdew–Burke–Ernzerhof exchange and correlation (XC) functional^[^ ^46^ ^]^ as implemented in Vienna Ab initio Simulation Package (VASP).^[^ ^47^ ^]^ The *T*‐dependent phonons and anharmonicity were obtained from AIMD simulations in which the interatomic force constants (AIMD‐IFCs) were extracted with the TDEP package.^[^ ^35^ ^]^ The particle‐like contribution was computed by solving the standard Peierls–Boltzmann transport equation (BTE) with the inclusion of 3ph and 4ph scatterings as implemented in ShengBTE package,^[^ ^24^, ^32^ ^]^ in which the AIMD‐IFCs were converted from TDEP format to ShengBTE format^[^ ^24^ ^]^ using in‐house scripts. The computation of the wave‐like contribution used the solution of Wigner transport equation^[^ ^39^ ^]^ as implemented in Phono3py package^[^ ^19^, ^25^ ^]^ which only considered 3ph scattering. For this work, an in‐house extension of Phono3py was developed to include 4ph scattering in the wave‐like contribution. More computational details are provided in Supporting Information.

### Statistical Analysis

Results are represented as means +/± SD.

## Conflict of Interest

The authors declare no conflict of interest.

## Supporting information

Supporting Information

## Data Availability

The data that support the findings of this study are available from the corresponding author upon reasonable request.
