# Supplementary material for: Predicting the Lattice Thermal Conductivity in Nitride Perovskite LaWN3 from ab initio Lattice Dynamics
Source: Adv Sci (Weinh). 2023 Jan 22;10(9):2205934. doi: 10.1002/advs.202205934 (PMC10037690; doi:10.1002/advs.202205934)
Supplement: Supplementary file 1 — Supporting Information [file ADVS-10-2205934-s001.pdf]

ADVANCED SCIENCE
Open Access

Supporting Information

for *Adv. Sci.*, DOI 10.1002/advs.202205934

Predicting the Lattice Thermal Conductivity in Nitride Perovskite $LaWN_3$ from ab initio Lattice Dynamics

Zhen Tong, Yatian Zhang, Alessandro Pecchia, ChiYung Yam, Liujiang Zhou, Traian Dumitrică* and Thomas Frauenheim**

Supplemental Material

Predicting the lattice thermal conductivity in nitride perovskite $LaWN_3$ from *ab initio* lattice dynamics

Zhen Tong,[1, 2, *] Yatian Zhang,[3] Alessandro Pecchia,[4] ChiYung Yam,[1]
Liujiang Zhou,[5] Traian Dumitrică,[6, †] and Thomas Frauenheim[1, 2, 3, ‡]

[1]*Shenzhen JL Computational Science and Applied Research Institute, Shenzhen 518131, China.*
[2]*Beijing Computational Science Research Center, Beijing 100193, China*
[3]*Bremen Center for Computational Materials Science, University of Bremen, Bremen 2835, Germany*
[4]*CNR-ISMN, Via Salaria km 29.300, Monterotondo 00017, Rome, Italy*
[5]*School of Physics, University of Electronic Science and Technology of China, Chengdu 610054, China*
[6]*Department of Mechanical Engineering, University of Minnesota, Minnesota 55455, United States of America*

CONTENTS

Density Functional Theory Calculations S1

Harmonic and Anharmonic Force Constants S1

Phonon Scattering Rates from Three- and Four-phonon Interactions S2

Supplemental Figures S3

References S5

DENSITY FUNCTIONAL THEORY CALCULATIONS

All *ab initio* calculations are carried out using projector-augmented-wave (PAW) [1] method with the Perdew-Burke-Ernzerhof exchange and correlation (XC) functional [2] as implemented in Vienna Ab initio Simulation Package (VASP) [3]. The La $5s^2 5p^6 5d^1 6p^2$, N $2s^2 2p^3$, W $5d^5 6s^1$ shells are treated as valence states. Kinetic energy cutoff value of 800 eV was used for the wavefunctions. The force and energy convergence thresholds of 10^{-6} eV/Å and 10^{-14} eV, respectively, were used for both structural relaxation and self-consistent density functional theory (DFT) calculations. The Brillouin zone was sampled with a Monkhorst–Pack mesh of 13×13×13 for the structure relaxation of $LaWN_3$ with a primitive cell containing 10 atoms for rhombohedral ($R3c$, Space Group 161) symmetry. The relaxed lattice constants are $a = 5.58$ Å and $\alpha = 60.49°$, which is within 1.00 % of the experimental values ($a = 5.64$ Å and $\alpha = 60.33°$[4]).

HARMONIC AND ANHARMONIC FORCE CONSTANTS

Ground Force Constants Calculations: The ground interatomic force constants (G-IFCs) are calculated at 0 K. The second-order G-IFCs^{2nd} have been computed on a 3×3×3 supercell with 3×3×3 **k**-points using density functional perturbation theory (DFPT) and accounting for the non-analytic term correction due to the dielectric tensor and Born effective charges, which are then extracted using Phonopy [5]. The third-order G-IFCs^{3rd} have been computed on a 2×2×2 supercell with Gamma **k**-points in which the diameter cutoff distance is limited to 0.7 nm, using finite-displacement approach as implemented in the THIRDORDER.PY script packaged in ShengBTE [6]. The fourth-order G-IFCs^{4th} have been computed on a 2×2×2 supercell with Gamma **k**-points in which the diameter cutoff distance is limited to 0.4 nm, using finite-displacement approach with our in-house code [7–9]. The shorter cutoff distance for the G-IFCs^{4th} stems from the earlier observation [10] that high-order IFCs are often more spatially localized, which has been further confirmed with previous works [8, 9, 11]. The particle-like component of the thermal conductivity (κ_c) formula in Eq. (2) of the main text has been computed using ShengBTE for 3ph [6] and its extension for 3+4ph [12]. In addition, the G-IFCs^{2nd} have been converted from Phonopy format to hdf5 format using phonopy [5], and G-IFCs^{3rd} have been converted to hdf5 format using hiphive [13]. The coherence

thermal conductivity (κ_c) formula in Eq. (3) of the main text has been implemented in the Phono3py package [14] by Simoncelli et al. [15]. We here developed an in-house extension of Phono3py to involve the 4ph scattering in computing the wave-like contribution. The thermal conductivity (κ_c and κ_p) based on G-IFCs has been computed based on a mesh 9×9×9 using the single-mode relaxation time approximation (SMA), since for ultralow thermal conductivity materials the exact Peierls-Boltzmann thermal conductivity is known to be practically indistinguishable from the SMA value [11, 15–17].

Temperature-dependent Force Constants Calculations: The temperature-dependent interatomic force constants (AIMD-IFCs) which are extracted from the *ab initio* molecular dynamics (AIMD) simulations as implemented in the TDEP package [18]. The Born-Oppenheimer molecular dynamics with the PAW [1] method as implemented in VASP [3] on a 4×4×4 supercell containing 640 atoms. The kinetic energy cutoff, the force, and the energy convergence thresholds are set to be 800 eV, 10^{-3} eV/Å and 10^{-5} eV, respectively. For the Brillouin zone integration, we use the Γ-point and ran the simulations on a grid of temperatures and volumes in the canonical ensemble. Temperature was controlled using a Langevin Thermostat [19]. The simulations ran for about 20 ps with a time step of 2 fs. When fitting the IFCs, the cutoffs were set to be 0.8 nm, 0.7 nm, and 0.4 nm for the AIMD-IFCs^{2nd}, AIMD-IFCs^{3rd}, and AIMD-IFCs^{4th}, respectively. The AIMD-IFCs have been converted from TDEP format to Phonopy format [5] and ShengBTE format [6] using in-house scripts. The thermal conductivity (κ_c and κ_p) based on the AIMD-IFCs (temperature-dependent phonon frequency and anharmonicity) has been computed based on a mesh 9×9×9.

PHONON SCATTERING RATES FROM THREE- AND FOUR-PHONON INTERACTIONS

Phonon scattering rates associated with the three- (Γ_λ^{3ph}) and four-phonon (Γ_λ^{4ph}) processes are all calculated using the renormalized phonon frequencies. The detailed expressions for Γ_λ^{3ph} and Γ_λ^{4ph} in the single mode relaxation time approximation (SMRTA) [20, 21] are below.

Three-phonon scattering rates:

$$\Gamma_\lambda^{3ph} = \sum_{\lambda_1\lambda_2}\left(\frac{1}{2}\Gamma_\lambda^{\lambda_1\lambda_2} + \Gamma_{\lambda\lambda_1}^{\lambda_2}\right), \tag{S1}$$

where $\Gamma_{\lambda\lambda_1}^{\lambda_2}$ and $\Gamma_\lambda^{\lambda_1\lambda_2}$ represent the absorption and emission scattering in three-phonon scattering processes rates and can be calculated as [21–23]

$$\Gamma_\lambda^{\lambda_1\lambda_2} = \frac{\pi\hbar}{4N}(1+n_{\lambda_1}^0+n_{\lambda_2}^0)\left|\Psi_-^{(3ph)}\right|^2\Delta_{\mathbf{k}}^{\mathbf{k}_1\mathbf{k}_2}\frac{\delta(\omega_\lambda-\omega_{\lambda_1}-\omega_{\lambda_2})}{\omega_\lambda\omega_{\lambda_1}\omega_{\lambda_2}}, \tag{S2}$$

$$\Gamma_{\lambda\lambda_1}^{\lambda_2} = \frac{\pi\hbar}{4N}(n_{\lambda_1}^0-n_{\lambda_2}^0)\left|\Psi_+^{(3ph)}\right|^2\Delta_{\mathbf{k}\mathbf{k}_1}^{\mathbf{k}_2}\frac{\delta(\omega_\lambda+\omega_{\lambda_1}-\omega_{\lambda_2})}{\omega_\lambda\omega_{\lambda_1}\omega_{\lambda_2}}, \tag{S3}$$

$$\Psi_\pm^{(3ph)} = \sum_{b,l_1b_1,l_2b_2}\sum_{\alpha\alpha_1\alpha_2}\Phi_{0b,l_1b_1,l_2b_2}^{\alpha\alpha_1\alpha_2}\frac{e_{\alpha b}^{\lambda}e_{\alpha_1b_1}^{\pm\lambda_1}e_{\alpha_2b_2}^{-\lambda_2}}{\sqrt{\bar{m}_b\bar{m}_{b_1}\bar{m}_{b_2}}}e^{\pm i\mathbf{k}_1\mathbf{r}_{l_1}}e^{-i\mathbf{k}_2\mathbf{r}_{l_2}}. \tag{S4}$$

$\Delta_{\mathbf{k}}^{\mathbf{k}_1\mathbf{k}_2}$ and $\Delta_{\mathbf{k}\mathbf{k}_1}^{\mathbf{k}_2}$ account for the momentum selection rules (Fermi's golden rule, FGR) for the absorption process $\mathbf{k} = \mathbf{k}_1 + \mathbf{k}_2 + \mathbf{R}$ and the emission process $\mathbf{k} + \mathbf{k}_1 = \mathbf{k}_2 + \mathbf{R}$ respectively, with the reciprocal lattice vector $\mathbf{R} = \mathbf{0}$ representing Normal (N) processes and $\mathbf{R} \neq \mathbf{0}$ representing Umklapp (U) processes. N is the total number of **k**-points or primitive cells, n^0 is the Bose-Einstein distribution for accounting the phonon occupation number, ω is the phonon frequency, and e is the phonon eigenvector. l, b, and α label the indexes of unit cells, basis atoms, and (x,y,z) directions, respectively. $\Phi_{0b,l_1b_1,l_2b_2}^{\alpha\alpha_1\alpha_2}$ is third-order interatomic force constants.

Four-phonon scattering rates:

$$\Gamma_\lambda^{4ph} = \left(\frac{1}{6}\sum_{\lambda_1\lambda_2\lambda_3}\Gamma_\lambda^{\lambda_1\lambda_2\lambda_3} + \frac{1}{2}\sum_{\lambda_1\lambda_2\lambda_3}\Gamma_{\lambda\lambda_1}^{\lambda_2\lambda_3} + \frac{1}{2}\sum_{\lambda_1\lambda_2\lambda_3}\Gamma_{\lambda\lambda_1\lambda_2}^{\lambda_3}\right), \tag{S5}$$

where $\Gamma_\lambda^{\lambda_1\lambda_2\lambda_3}$, $\Gamma_{\lambda,\lambda_1}^{\lambda_2\lambda_3}$, and $\Gamma_{\lambda\lambda_1\lambda_2}^{\lambda_3}$ account for the absorption, redistribution, and emission scattering rates in four-

phonon scattering processes and can be obtained as [21–23]

$$\Gamma_{\lambda}^{\lambda_1\lambda_2\lambda_3} = \frac{\pi\hbar}{4N}\frac{\hbar}{2N}\frac{n^0_{\lambda_1}n^0_{\lambda_2}n^0_{\lambda_3}}{n^0_{\lambda}}\left|\Psi^{(4\mathrm{ph})}_{--}\right|^2 \Delta_{\mathbf{k}}^{\mathbf{k_1k_2k_3}}\frac{\delta(\omega_\lambda-\omega_{\lambda_1}-\omega_{\lambda_2}-\omega_{\lambda_3})}{\omega_\lambda\omega_{\lambda_1}\omega_{\lambda_2}\omega_{\lambda_3}}, \tag{S6}$$

$$\Gamma_{\lambda\lambda_1}^{\lambda_2\lambda_3} = \frac{\pi\hbar}{4N}\frac{\hbar}{2N}\frac{(1+n^0_{\lambda_1})n^0_{\lambda_2}n^0_{\lambda_3}}{n^0_{\lambda}}\left|\Psi^{(4\mathrm{ph})}_{+-}\right|^2 \Delta_{\mathbf{kk_1}}^{\mathbf{k_2k_3}}\frac{\delta(\omega_\lambda+\omega_{\lambda_1}-\omega_{\lambda_2}-\omega_{\lambda_3})}{\omega_\lambda\omega_{\lambda_1}\omega_{\lambda_2}\omega_{\lambda_3}}, \tag{S7}$$

$$\Gamma_{\lambda\lambda_1\lambda_2}^{\lambda_3} = \frac{\pi\hbar}{4N}\frac{\hbar}{2N}\frac{(1+n^0_{\lambda_1})(1+n^0_{\lambda_2})n^0_{\lambda_3}}{n^0_{\lambda}}\left|\Psi^{(4\mathrm{ph})}_{++}\right|^2 \Delta_{\mathbf{kk_1k_2}}^{\mathbf{k_3}}\frac{\delta(\omega_\lambda+\omega_{\lambda_1}+\omega_{\lambda_2}-\omega_{\lambda_3})}{\omega_\lambda\omega_{\lambda_1}\omega_{\lambda_2}\omega_{\lambda_3}}, \tag{S8}$$

$$\Psi^{(4\mathrm{ph})}_{\pm\pm} = \sum_{b,l_1b_1,l_2b_2,l_3b_3}\sum_{\alpha\alpha_1\alpha_2\alpha_3}\Phi^{\alpha\alpha_1\alpha_2\alpha_3}_{0b,l_1b_1,l_2b_2,l_3b_3}\frac{e^{\lambda}_{\alpha b}e^{\pm\lambda_1}_{\alpha_1b_1}e^{\pm\lambda_2}_{\alpha_2b_2}e^{-\lambda_3}_{\alpha_3b_3}}{\sqrt{\bar{m}_b\bar{m}_{b_1}\bar{m}_{b_2}\bar{m}_{b_3}}}e^{\pm i\mathbf{k_1}\cdot\mathbf{r}_{l_1}}e^{\pm i\mathbf{k_2}\cdot\mathbf{r}_{l_2}}e^{-i\mathbf{k_3}\cdot\mathbf{r}_{l_3}}, \tag{S9}$$

$\Delta_{\mathbf{k}}^{\mathbf{k_1k_2k_3}}$, $\Delta_{\mathbf{kk_1}}^{\mathbf{k_2k_3}}$, and $\Delta_{\mathbf{kk_1k_2}}^{\mathbf{k_3}}$ specify the momentum selection rules (Fermi's golden rule, FGR) for the absorption process $\mathbf{k}=\mathbf{k}_1+\mathbf{k}_2+\mathbf{k}_3+\mathbf{R}$, redistribution process $\mathbf{k}+\mathbf{k}_1=\mathbf{k}_2+\mathbf{k}_3+\mathbf{R}$, and emission process $\mathbf{k}+\mathbf{k}_1+\mathbf{k}_2=\mathbf{k}_3+\mathbf{R}$, respectively, with the reciprocal lattice vector $\mathbf{R}=\mathbf{0}$ representing Normal (N) processes and $\mathbf{R}\neq\mathbf{0}$ representing Umklapp (U) processes. $\Phi^{\alpha\alpha_1\alpha_2\alpha_3}_{0b,l_1b_1,l_2b_2,l_3b_3}$ is fourth-order interatomic force constants.

SUPPLEMENTAL FIGURES

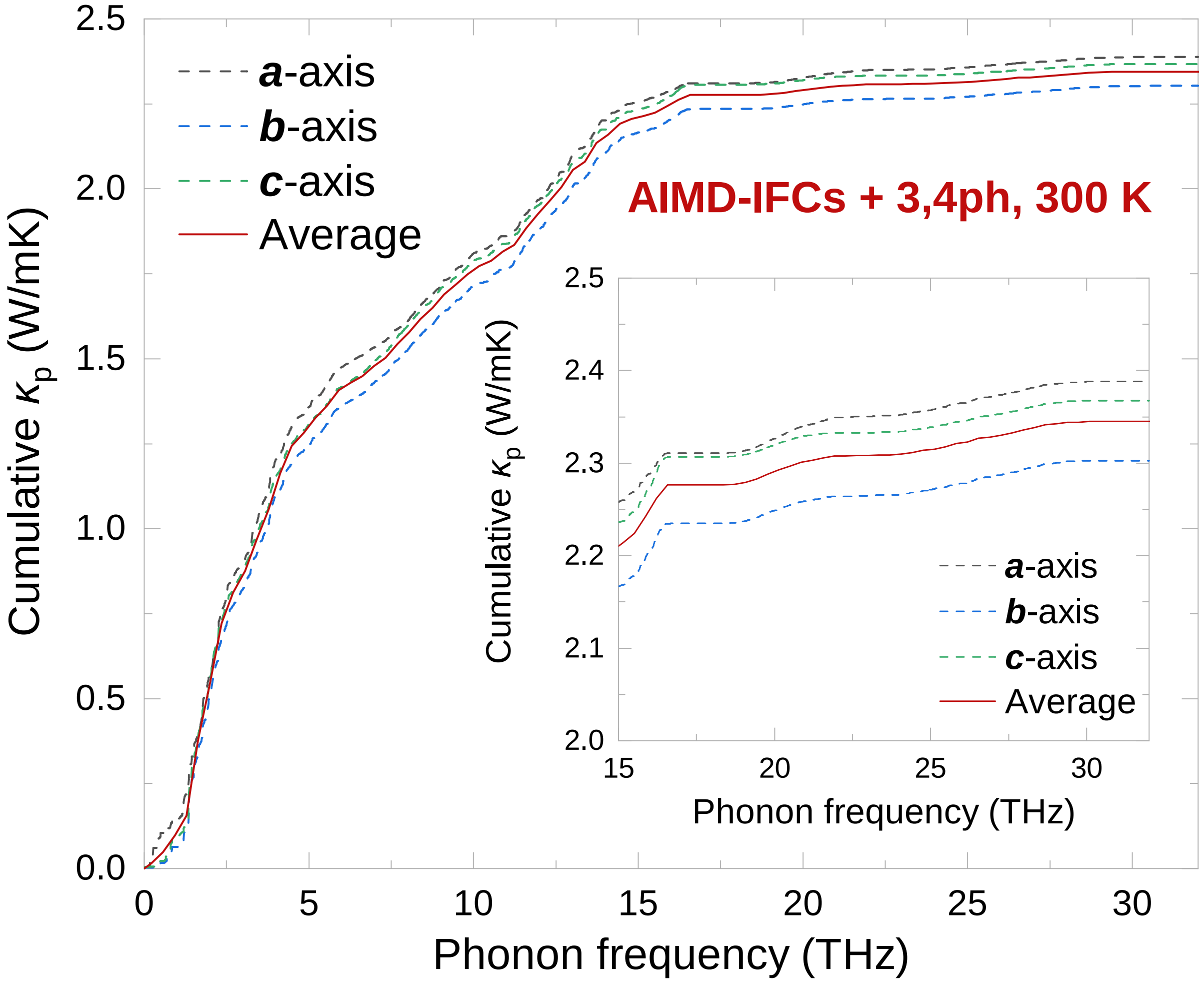

Fig. S1. Cumulative thermal conductivity κ_p of $LaWN_3$ along the different crystallographic directions at 300 K under the theory level of AIMD-IFCs.

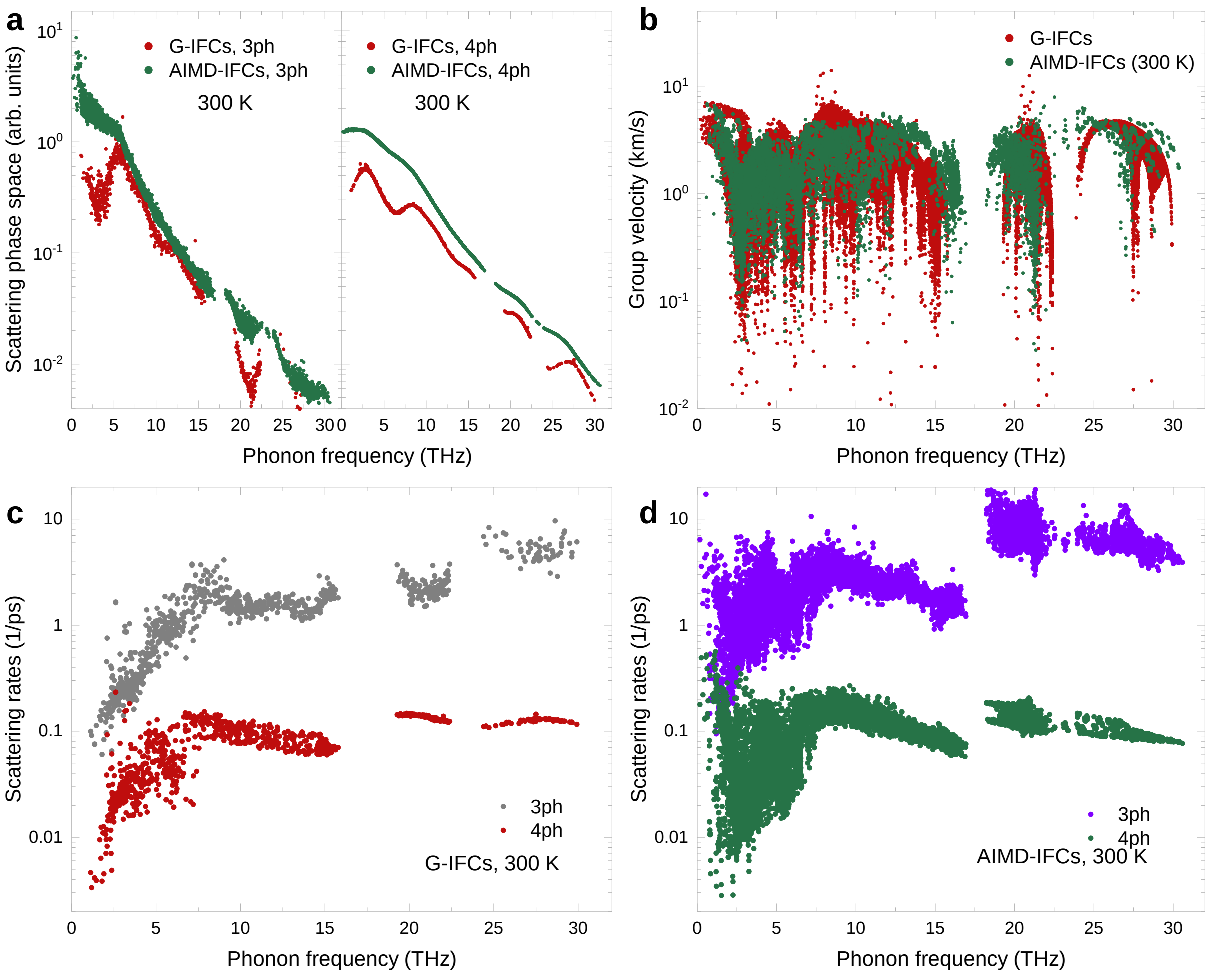

Fig. S2. (a) Phonon scattering phase space of $LaWN_3$ at 300 K for 3 and 4ph scatterings with G-IFCs and AIMD-IFCs. (b) Phonon group velocity calculated with G-IFCs and AIMD-IFCs. (c) The 3ph and 4ph scattering rates at 300 K for G-IFCs. (d) Same as (c) but for AIMD-IFCs.

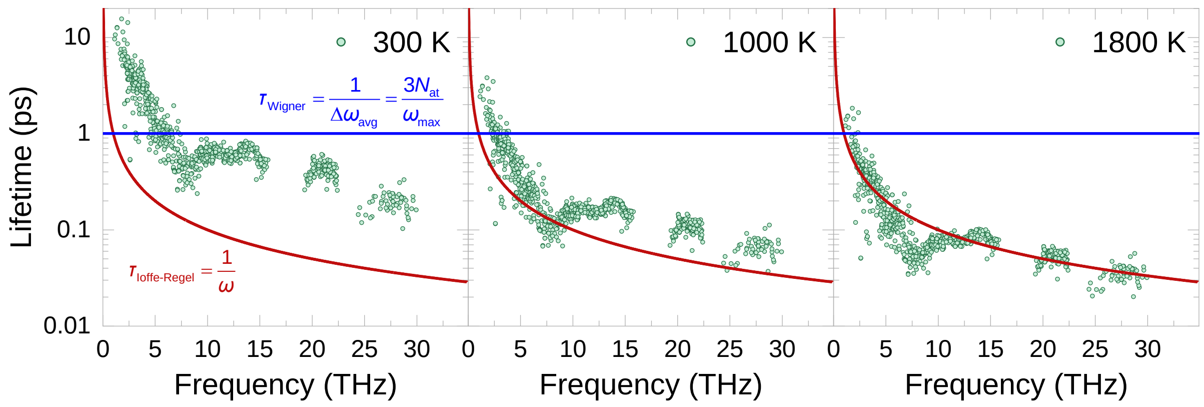

Fig. S3. Phonon lifetime due to 3+4ph interactions based on G-IFCs at 300 K, 1000 K and 1800 K.

* tongzhen@csar.ac.cn
† dtraian@umn.edu
‡ thomas.frauenheim@bccms.uni-bremen.de

[1] G. Kresse and D. Joubert, From ultrasoft pseudopotentials to the projector augmented-wave method, Phys. Rev. B **59**, 1758 (1999).
[2] J. P. Perdew, K. Burke, and M. Ernzerhof, Generalized Gradient Approximation Made Simple, Phys. Rev. Lett. **77**, 3865 (1996).
[3] G. Kresse and J. Hafner, *Ab initio* molecular dynamics for liquid metals, Phys. Rev. B **47**, 558 (1993).
[4] K. R. Talley, C. L. Perkins, D. R. Diercks, G. L. Brennecka, and A. Zakutayev, Synthesis of $LaWN_3$ nitride perovskite with polar symmetry, Science **374**, 1488 (2021).
[5] A. Togo and I. Tanaka, First principles phonon calculations in materials science, Scr. Mater. **108**, 1 (2015).
[6] W. Li, J. Carrete, N. A. Katcho, and N. Mingo, ShengBTE: A solver of the Boltzmann transport equation for phonons, Comput. Phys. Commun. **185**, 1747 (2014).
[7] Z. Tong, X. Yang, T. Feng, H. Bao, and X. Ruan, First-principles predictions of temperature-dependent infrared dielectric function of polar materials by including four-phonon scattering and phonon frequency shift, Phys. Rev. B **101**, 125416 (2020).
[8] Z. Tong, T. Dumitrică, and T. Frauenheim, Ultralow Thermal Conductivity in Two-Dimensional MoO $_3$, Nano Letters **21**, 4351 (2021).
[9] Z. Tong, A. Pecchia, C. Yam, H. Bao, T. Dumitrică, and T. Frauenheim, Significant Increase of Electron Thermal Conductivity in Dirac Semimetal Beryllonitrene by Doping Beyond Van Hove Singularity, Adv. Funct. Mater. **32**, 2111556 (2022).
[10] F. Zhou, W. Nielson, Y. Xia, and V. Ozoliņš, Lattice Anharmonicity and Thermal Conductivity from Compressive Sensing of First-Principles Calculations, Phys. Rev. Lett. **113**, 185501 (2014).
[11] Y. Xia, K. Pal, J. He, V. Ozolins, and C. Wolverton, Particlelike Phonon Propagation Dominates Ultralow Lattice Thermal Conductivity in Crystalline Tl 3 VSe 4, Phys. Rev. Lett. **124**, 065901 (2020).
[12] Z. Han, X. Yang, W. Li, T. Feng, and X. Ruan, FourPhonon: An extension module to ShengBTE for computing four-phonon scattering rates and thermal conductivity, arXiv:2104.04895 [cond-mat] (2021).
[13] F. Eriksson, E. Fransson, and P. Erhart, The Hiphive Package for the Extraction of High-Order Force Constants by Machine Learning, Adv. Theory Simul. **2**, 1800184 (2019).
[14] A. Togo, L. Chaput, and I. Tanaka, Distributions of phonon lifetimes in Brillouin zones, Phys. Rev. B **91**, 094306 (2015).
[15] M. Simoncelli, N. Marzari, and F. Mauri, Unified theory of thermal transport in crystals and glasses, Nat. Phys. **15**, 809 (2019).
[16] Y. Luo, X. Yang, T. Feng, J. Wang, and X. Ruan, Vibrational hierarchy leads to dual-phonon transport in low thermal conductivity crystals, Nat. Commun. **11**, 2554 (2020).
[17] A. Jain, Multichannel thermal transport in crystalline Tl_3VSe_4, Phys. Rev. B **102**, 201201 (2020).
[18] O. Hellman and I. A. Abrikosov, Temperature-dependent effective third-order interatomic force constants from first principles, Phys. Rev. B **88**, 144301 (2013).
[19] W. G. Hoover, A. J. C. Ladd, and B. Moran, High-Strain-Rate Plastic Flow Studied via Nonequilibrium Molecular Dynamics, Phys. Rev. Lett. **48**, 1818 (1982).
[20] A. A. Maradudin and A. E. Fein, Scattering of Neutrons by an Anharmonic Crystal, Phys. Revi. **128**, 2589 (1962).
[21] G. D. Mahan, Many-Particle Physics, 3rd ed. (Kluwer Academic/Plenum Publishers, New York, 2000).
[22] L. Lindsay, D. A. Broido, and N. Mingo, Lattice thermal conductivity of single-walled carbon nanotubes: Beyond the relaxation time approximation and phonon-phonon scattering selection rules, Phys. Rev. B **80**, 125407 (2009).
[23] T. Feng and X. Ruan, Quantum mechanical prediction of four-phonon scattering rates and reduced thermal conductivity of solids, Phys. Rev. B **93**, 045202 (2016).
